# *“Nobody knows, or seems to know how rheumatology and breastfeeding works”*: Women's experiences of breastfeeding whilst managing a long-term limiting condition – A qualitative visual methods study

**DOI:** 10.1016/j.midw.2019.08.002

**Published:** 2019-11

**Authors:** Denitza Williams, Jessica Webber, Bethan Pell, Aimee Grant, Julia Sanders, Ernest Choy, Adrian Edwards, Ann Taylor, Meng-Chieh Wu, Rhiannon Phillips

**Affiliations:** a Division of Population Medicine, School of Medicine, Cardiff University, Cardiff, UK; b Centre for Trials Research, Cardiff University, Cardiff, UK; c School of Healthcare Sciences, Cardiff University, Cardiff, UK; d Division of Infection and Immunity, School of Medicine, Cardiff University, Cardiff, UK; e Centre for Medical Education, Cardiff University, Cardiff, UK

**Keywords:** Breastfeeding, Qualitative, Autoimmune rheumatic disease, Long-term illness, Disability, Shared decision-making, Timeline-facilitated interview, Time-lining, Visual methods

## Abstract

**Background:**

Only around 1% of babies in the UK are breastfed exclusively until six months of age as recommended by the World Health Organisation. One in ten women who have recently given birth in the UK have a long-term illness and they are at increased risk of stopping breastfeeding early. We considered women with autoimmune rheumatic diseases as an exemplar group of long term illnesses, to explore the barriers and enablers to breastfeeding

**Aim:**

To understand the experiences of infant feeding among women with autoimmune rheumatic diseases and to identify potential barriers and enablers.

**Design:**

Qualitative visual timeline-facilitated interviews.

**Participants and setting:**

128 women with autoimmune rheumatic diseases who were considering pregnancy, pregnant, or had young children took part in an online survey as part of the STAR Family Study. Of these, 13 women who had children were purposefully sampled to be interviewed. Interviews took place in person or on the telephone. Timeline-facilitated interviews were used to focus on lived experiences and topics important to the women, including early parenting. We conducted a focused thematic analysis of women's lived experiences of infant feeding.

**Results:**

Three main themes were identified in relation to breastfeeding: lack of information about medication safety, lack of support in decision-making and maintaining breastfeeding, and maternal guilt.

**Conclusions:**

Women with autoimmune rheumatic diseases found it difficult to access the information they needed about medications to make informed decisions about breastfeeding. They often also felt pressurised into breastfeeding and experienced feelings of guilt if they were unable, or did not wish to breastfeed. Tailored interventions are required that adopt a non-judgmental and person-centred approach to support decision-making in regard to infant feeding, providing women with information that can best enable them to make infant feeding choices.

## Introduction

The World Health Organization recommends exclusive breastfeeding for infants for six months in order to allow optimal growth and development as well as improving maternal health outcomes (Organization, W.H. 2002). Initiation of breastfeeding has increased in the U.K. for the past decade, but the number of babies being breastfed exclusively at six months has not increased (McAndrew et al., 2012; Pokhrel et al., 2015). It has been found that although 81% of babies in U.K. are breastfed at least once (McAndrew et al., 2012), only 23% are breastfed exclusively by six weeks, and only 1% until 6 months (Bolling et al., 2007). women with and without physical disabilities have comparable rates of pregnancy in general (Iezzoni et al., 2013), and breastfeeding is recommended for all women (Smeltzer, 2007), whether or not they have physical disability. Many women report living with a long-term illness. In fact, almost one in ten (9.4%) women who have recently given birth in the UK have a limiting long-term illness (Sumilo et al., 2012).

The current healthcare context places extra pressure on women to breastfeed, with the World Health Organization recommending a hierarchy of infant feeding from 1. Breastfeeding, 2. Breastmilk from a donor and then 3. Formula feeding. However, women with long-term illnesses are significantly less likely to initiate and maintain breastfeeding than women without a long-term illness (Sumilo et al., 2012; Malouf et al., 2017; Redshaw et al., 2013; Morton et al., 2013; Mitra et al., 2015). Furthermore, the U.K. does not have a network of donor banks that women with long-term conditions can access. Therefore, women might benefit from more targeted and tailored support with infant feeding.

Qualitative studies have found that mothers with long-term conditions struggle to breastfeed their infants for the recommended six months (Schaefer, 2004). A study looking at breastfeeding amongst women with physical disabilities highlighted multiple barriers to breastfeeding, including medication considerations, limited information specific to their condition, lack of support, and difficulties with milk supply and latching (Powell et al., 2017). Women have also reported a lack of information about the safety of medications when breastfeeding relating to the management of probable post-partum flares of disease activity (Briggs et al., 2016). Women with fibromyalgia found initiating and sustaining breastfeeding difficult and frustrating due to the pain and fatigue associated with their long-term condition (Schaefer, 2004). However, a range of facilitators were identified, including physical help with breastfeeding from others such as a spouse, adaptive strategies and equipment, the use of a breast pump, and peer support from other women with physical disabilities (Powell et al., 2017).

Women of reproductive age who have an Autoimmune Rheumatic Disease (ARD), such as Lupus, inflammatory (rheumatoid) arthritis (RA), or vasculitis will be used as an exemplar group in this paper to highlight the infant feeding experiences of women with a long-term condition. Similar to other women with long-term conditions, women with an ARD face a range of difficulties with pregnancy planning, pregnancy, and early parenting due to fluctuation in disease activity, treatments for their disease, and difficulties with physical functioning, pain, and fatigue (Ackerman et al., 2015; Phillips et al., 2018b; Ostensen and Foerger, 2013; Phillips et al., 2018a). A recent survey with women who have an ARD identified a need for more breastfeeding information (Phillips et al., 2018a). Many women who have an ARD will actually be able to breastfeed whilst also managing their long-term condition, especially with the availability of newer medications and better evidence coming through regarding the safety of breastfeeding whilst on certain medications (Flint, 2016). However, this needs to be balanced with the fact that some women may not want to or feel able to breastfeed. Although, the physical and emotional health benefits of breastfeeding for babies and mothers are unequivocal, we must ensure that all care is mother and family centred, supportive and non-judgemental and that mothers decisions are supported and respected (Briggs et al., 2016; UNICEF). This study aims to understand the lived experiences of women with ARDs to identify potential barriers and enablers to breastfeeding and to identify elements of a tailored intervention

## Methods

This study forms part of the STAR family study (Phillips et al., 2018a) which sought to understand the experiences of women with autoimmune rheumatic diseases who were thinking about starting a family, were pregnant or had young children. STAR family study used mixed-methods, which included an online cross-sectional survey and qualitative interviews with women with ARDs and healthcare professionals (Phillips et al., 2018a).

In this manuscript, we report on a focused analysis of qualitative timeline-facilitated, narrative interviews with women with ARDs who had children, to examine in-depth their lived experiences of infant feeding. Survey results and healthcare professional views of preconception, pregnancy and postpartum care of women with ARDs are reported elsewhere (Phillips et al., 2018a).

### Participants and recruitment

Women were purposively sampled from the STAR Family study survey for the qualitative interviews (Phillips et al., 2018a). The survey was completed by 128 women aged 18–49 years, living in the UK, who have an ARD and were either; considering having children, pregnant, or had at least one child under 5 years of age. Women who had consented to being contacted for a possible interview through the survey were contacted via telephone or e-mail. Women who expressed interest following contact were sent a participant information pack containing participant information sheet, consent form and stamped return envelope. To ensure as many interviews took place face-to-face as possible, women within two hours travelling distance of Cardiff University (South Wales, UK) were initially contacted. The sampling was then expanded to include women across the UK who were willing to take part in telephone interviews. Purposive sampling strategy was applied to include an equal representation of women who were either planning a pregnancy, currently pregnant or already had children. Women were provided with a £20 shopping voucher as a thank you for taking part. The sub-set of interviews with women who already had children were selected for this focused analysis of infant feeding experiences.

### Interview procedure

Interviews took place either at the women's home *N* = 4), or over the telephone (*n* = 9). Interviews were conducted by DW (PhD) and BP (BSc). DW is a post-doctoral researcher with a specific interest in women's health and BP was a research assistant with experience in qualitative methods at the time of the interviews. Both interviewers were female and had previous experience in conducting qualitative interviews and were provided with training and guidance in timeline assisted interviews by the qualitative lead and the principal investigator. When considering positionality, DW was a mother with young children, whilst BP was childless at the time of the interviews. The interviewers had no relationship with the participants and had no prior knowledge of their breastfeeding experiences. For pragmatic reasons, babies and children were sometimes present during the interviews, but no other adults were present.

We adopted a person-centred approach to interviewing, using timeline-facilitated narrative interviews (Goldenberg et al., 2016) . Timelines have been shown to be useful in life story research because they can facilitate reflection of personal experience within a wider context (Adriansen, 2012). They can also be helpful to build a better participant interviewer rapport by addressing power imbalance, and encourage a richer narrative by reinforcing ownership of personal stories (Sheridan et al., 2011). The objective was to facilitate women to reflect on their own lived experiences of breastfeeding highlighting the importance of using timeline-facilitated interviews. Therefore, instead of a topic guide, we used a flexible narrative approach to encourage women to focus on topics important to them, rather than being prompted by researcher-generated topics.

Women were encouraged to lead the interview and talk about their ‘lived experiences’ in their own words, by using the timeline as an *aide memoire*, rather than being guided by an interview schedule (Flint, 2016), in order to build rapport and address power imbalance (Goldenberg et al., 2016). A resource pack was sent to the women before the interview which included a colourful ‘What To Expect’ sheet providing guidance on the types of topics which were of interest to the research team (Supplementary File 1) along with stationary items such as coloured highlighters, emoji stickers and an exemplar blank timeline. The women were told they could use other methods to create notes or prompts for themselves if they wished. The timelines provided a visual tool to enable women to map out their journey through pre-conception, pregnancy and infant feeding, noting key events and their physical and emotional responses to these. We describe our use of timelines in this study more detail in a separate methodological paper (Pell et al., 2019).

### Analysis

Interviews were audio-recorded and transcribed verbatim. The data were analysed using Braun and Clarke's approach to thematic analysis (Braun and Clarke, 2006), which included familiarising with the data, generating initial codes, searching for themes, reviewing and refining themes and defining and naming themes. These were discussed at regular team meetings. An inductive approach was used in analysis to allow themes to emerge from the data in order to understand the topics most important to the women interviewed. Data were not double coded, instead themes were discussed in qualitative team meetings to discuss data production, the development of the coding framework and data analysis. This approach has been identified as appropriate in qualitative research (Barbour, 2001). We were guided by the concept of ‘information power’ (Malterud et al., 2016) rather than ‘saturation’; NVIVO 11 software was used to organise the data.

## Results

The STAR family study online survey was completed by 128 women. Twenty-two women who had expressed interest to be contacted for an interview were interviewed, 13 of whom already had children and were therefore eligible for inclusion in this study.

The women interviewed (*n* = 13) reported a wide range of ARDs including inflammatory arthritis (e.g. RA, psoriatic arthritis), systemic lupus erythematosus, and dermatomyositis. They had from one to three children who were between the ages of 6 weeks and 17 years old (see Table 1). Women discussed a large range of topics covering various aspects of parenting which have been reported elsewhere (Phillips et al., 2018a). These included adaptations they had made to their parenting because of their ARD; the desire to give their child ‘normal’ experiences; physical problems they experienced, especially coping with post-partum flares of their conditions; their identity as a parent and as an individual during this time; mental health problems; and birth experiences (Phillips et al., 2018a). In this manuscript, we focus on the themes relating to infant feeding.

**Table 1:** Participant characteristics.

Participant number	Condition	Children	Age	Number of children and age
1	Systemic Lupus Erythematosus	Yes	37	One child 1 year
2	Vasculitis	Yes	34	Two children 9 years, 1.5 years
3	Dermatamyositis	Yes	40	One child 1 year
4	Psoraitic arthritis	Yes	40	One child 1 year
5	Systemic Lupus Erythematosus	Yes	38	Three children 17 years,6 years,4 years
6	Idiopathic Juvenile Arthritis	Yes	37	One child 4 years
7	Inflammatory arthritis	Yes	37	Two children Twins 3 years old
8	Systemic Lupus Erythematosus	Yes	34	Three children 11 years,7 years,4 years
9	Psoriatic Arthritis	Yes	34	Two children 4 months, 2 years
10	Antisynthetase syndrome	Yes	32	One child 6 weeks
11	Rheumatoid Arthritis	Yes	40	Two children 2 years, 7 years
12	Rheumatoid Arthritis	Yes	30	Two children 3 years,1 year
13	Sjogren's syndrome	Yes	41	One child 2 years

One of the most prominent themes was a lack of information regarding the safety of medication in breastmilk. This was discussed by most of the women, whether they breastfed or not. The other major themes identified were; a lack of support regarding starting or stopping breastfeeding; and feelings of maternal guilt.

### Lack of information

Most women reported wanting to breastfeed their babies, and some felt that breastfeeding might have a protective impact on their children's susceptibility to rheumatological disorder later in life.

“Well breastfeeding was really important to me because I wanted to give, I wanted to give their immune system the best start in case well you know there's bound to be a hereditary component to, to all of this so I wanted to give them the best chance of not developing anything” (P4, 1 child)

However, women reported a lack of information regarding breastfeeding and ARDs. Women expressed concerns about the safety of medication during breastfeeding, and lack of shared decision- making when initiating or stopping breastfeeding.

Women felt that the information available regarding the safety of drugs used for managing ARDs whilst breastfeeding was limited and inconsistent. Some women felt that they were provided with contradictory information about the safety of drugs during breastfeeding from different health professionals, such as their GP, rheumatologist and midwife. Women discussed how information regarding the safety of some rheumatological medications in breastmilk was not available; for some drugs information was not available. For example, women might specifically ask their rheumatologist if they could breastfeed whilst on a certain medication and they would not provide an answer due to either lack of knowledge or information on the safety of a drug. Below, participant 9 describes how she was provided with inconsistent information about the effect of breastfeeding on her condition.

“The big thing I struggled with is that nobody knows, or seems to know how rheumatology and breastfeeding works and nobody seems to know about the sub-luxing and rheumatology, so when you are breastfeeding half the people told me I was sub-luxing more and having problems because I was breastfeeding, the other half said not […] I'd just get throwaway comments from the rheumatology team, well we'd like to put you on these medications, and I said can I co-sleep, can I breastfeed my child?” (P9, 2 children)

‘Sub-luxing’ can be painful and happens when a connecting bone is partially out of joint.

### Lack of support

Another theme discussed by many women was a general lack of support and empathy regarding breastfeeding from medical professionals. Different women discussed having felt pressured into initiating or continuing breastfeeding when they did not feel it was the best thing for them or their child. Although breastfeeding can have significant benefits for both mother and child, the choice to breastfeed is preference specific. A lack of patient-centred care and decision support regarding infant feeding options by some healthcare professionals was evident.

“I was so desperate to do what I'd thought what was right with these two tiny little babies, but actually as soon as I did give up breastfeeding… suddenly being a mum was so much more fun and I had two much happier babies because I was much more settled – and that's the thing I think of my journey that really kicks out as where it all went wrong.”

P7, 2 children

Some women felt that they needed to trade-off effective disease management with providing the best possible nutrition for their child.

Other women reflected on the often unacknowledged physical challenges associated with holding a baby in a breastfeeding position when they had musculoskeletal pain and the impact that had on their ability to further hold and interact with the baby.

*“My back was so bad I couldn't I had to put him down the minute I stopped feeding him so I didn't get any cuddles (laughs) not really you know I didn't”* (P4, 1 child)

Some women acknowledged that midwives focus on breastfeeding targets and hospital initiatives, but felt that although studies show the benefits of breastfeeding, they should look at what is best for each individual mother.

*“There is such a drive especially in the north-west, I think breastfeeding is really quite poor around here, there is such a drive to push you to breastfeed as it is best for the baby and I accepted it, but I also accept that sometimes the best thing in a research report isn't the best thing for the individual and in our case me and my two little ones it certainly wasn't and I think that was why in terms of the health visitor and the midwife and the breastfeeding advocates they're so hell bent on getting a twin mum to breastfeed, because it's that extra pressure again that they focus so much on that they forget the rest of the situation”* (P7, 2 children)

Conversely, several other mothers talked about how they felt pressured to stop breastfeeding, either by their healthcare professionals or by family members. This was sometimes driven by the need to start back on medication that may have been harmful to their baby. There was a general perception that breastfeeding places a strain and burden on women in terms of increasing their fatigue and delaying onset of certain medications.

Women reflected on a perceived contradiction between the advice of different healthcare professionals and that of different organisations such as the World Health Organisation's (WHO) breastfeeding guidelines.

*“I don't know how many people with rheumatology breastfeed like have they certainly didn't like the fact that I breastfed for more than a year, [child1] was fed until he self-weaned just before [child2] was born so he was 2½ but the WHO guidance says you should feed them until they're 2 but it's not socially acceptable, so rheumatology (…) they seem to frown on it a lot* (p9, 2 children)

*Family members’ concerns about the impact breastfeeding might have on mothers’ well-being was cited as a reason for advising women to stop breastfeeding:*

“[I was] pressured to stop breastfeeding… [family member though I was] wearing myself into the ground” P22

### Feelings of maternal guilt

The final theme found in discussions about breastfeeding was guilt. Many women felt guilty about their inability to breastfeed, for example if they had to stop breastfeeding in order to re-start medication, or their ability to parent whilst breastfeeding. Women reflected on the mismatch between their perception of what they ought to do, (‘ought self’) and their actual self (Higgins, 1987; Morley, 2010) in regards to being able to breastfeed their child and the impact their ARD had on that ability.

“A mind-set of guilt, that if you don't exclusively breastfeed your baby then you're the worst mother in the world… I was terribly guilty about it” (P22, 1 child)

Women's feeling of guilt seemed to stem in part from some healthcare professional and peer negative attitudes towards formula feeding. Women reported feeling guilty and ashamed of their desire not to breastfeed, inability to breastfeed or their decision to stop breastfeeding. Women wanted healthcare professionals to be more aware of the challenges they were facing having a young baby, breastfeeding and managing their ARD. They reflected on key turning points within their breastfeeding journey, such as postpartum disease flares.

“*The breastfeeding advocates had been trying to get me to consider alternative medication and stuff which in an ideal world when we have lots of time maybe that would be a nice thing to do, but when things go downhill drastically you know and I was in a lot of pain and I was struggling to hold 2 babies to breastfeed as well because my joints were sore… I marched on and then at 6 weeks I dropped a child”* (P7, 2 children)

The three themes were inherently interrelated. The lack of information on medication use in breastfeeding meant mothers were often in pain and unable to physically and sometimes psychologically parent to the best of their ability due to conservative medication management. This often led to guilt around their ability to parent, or if they stopped breastfeeding in order to start the medication, they felt guilty about prioritising their own health. Healthcare professionals’ attitudes towards formula-feeding meant many women felt shamed for their inability to, or choice not to breastfeed, which perpetuated their feelings of guilt. Whilst other women felt they were pressured to stop breastfeeding to accommodate optimal disease management.

## Discussion

Our findings provide new evidence about the range of barriers to breastfeeding that women with an ARD experience in the UK which influence their decisions about whether to breastfeed, and the duration of time to breastfeed their child. Women with an ARD often struggled to maintain breastfeeding whilst achieving optimum disease control. Most women reported that they felt pressure to breastfeed, whilst some also reported that they felt pressure to discontinue breastfeeding in order to re-instate certain medications. Overall, women felt that they needed more information about breastfeeding and medication, reported a greater need for support as well as feelings of guilt if they were unable or chose not to breastfeed.

Our findings about a lack of information regarding the safety of medication and breastfeeding are consistent with previous qualitative studies with women with long-term illnesses is the UK, the US and Australia, all of which indicate medication safety is a key factor contributing to a woman's decision to breastfeed (Powell et al., 2017; Webber et al., 2018; Payne and McPherson, 2010). The British Society of Rheumatology's guideline on prescribing drugs in breastfeeding indicates evidence about the safety of rheumatological medication in breastfeeding is limited or non-existent, especially for biological therapies (Flint et al., 2016). More research and correct signposting to resources is needed to be able to provide women with good quality, reliable data on the safety of rheumatological medications in breastfeeding as well as more education on this topic for women, rheumatologists, GPs, midwives, obstetricians and health visitors. Signposting to some of the specialist resources that are available for both women and healthcare professionals is needed. For example, websites such as the Breastfeeding Network (https://www.breastfeedingnetwork.org.uk/detailed-information/drugs-in-breastmilk/) and the Breastfeeding in Medication (http://www.breastfeeding-and-medication.co.uk/), provide information for both women and healthcare professionals about the safety of medications in breastmilk.

Women in our study discussed feeling pressurised to breastfeed, but not being adequately supported in doing so and sometimes not being able to do so, which caused tensions. Some women struggled with the physical demands of breastfeeding such as holding the baby in position as well as managing fatigue. Previous research has found that adaptive strategies and equipment can assist women with the physical demands of breastfeeding (Rogers, 2006). However, supportive equipment is rarely available and participants in this study did not report knowledge about or availability of such equipment. Previous studies in women with physical disabilities have reported similar challenges because their healthcare professionals and lactation consultants lacked information about how breastfeeding might interact with their disability, as well as guidance on adaptive strategies (Powell et al., 2017).

The fairly universal feelings of guilt about not breastfeeding amongst our interviewees have been found in previous studies in the general population (Guyer et al., 2013) and women with other long-term conditions (Tariq et al., 2016). A qualitative study looking at experiences of breastfeeding in healthy women found that guilt was most commonly felt when mothers perceived “that they had allowed their own needs to predominate over their infant's needs” (Guyer et al., 2013). Moreover, the culture around breastfeeding means that women can find that not doing so compromises their identity as a mother, whilst also causing concern about the impact not breastfeeding will have on the wellbeing of their child (Tariq et al., 2016).

This study has identified that women with ARDs have additional concerns about medication in breastmilk, managing fatigue, pain, and their ability to physically hold the baby in a breastfeeding position. There is a clear need for the incorporation and promotion of person-centred care when it comes to breastfeeding; a one size-fits all approach is not fit for purpose. Studies focusing on breastfeeding support indicate that women want to be listened to, not judged and not pressurised to breastfeed (Phillips, 2018c), and this is likely to be particularly important for women with ARDs where their illness can complicate their breastfeeding choices. To increase breastfeeding among women with ARDs and women with long-term conditions in general, healthcare providers, including lactation consultants and midwives who are traditionally thought to be proficient in breastfeeding knowledge, should be specifically trained about how they can support women with long-term conditions to breastfeed. Specifically, healthcare professionals need further training on the impact of having an ARD (or any chronic condition) can have on early parenting, the medications mothers might be taking, and the influence these factors might have on a women's ability to breastfeed. Although there are guidelines available for the pharmacological management of ARDs in women before, during and after pregnancy, healthcare professionals need to be trained in how to adequately support women or signpost to relevant information. Supporting women with long-term conditions will require patient-centred discussions around medication and infant feeding, the pros and cons of breastfeeding for women's well-being and disease management, the availability of peer-support, as well as strategies to facilitate positioning and fatigue. The incorporation of a shared decision-making approach could ensure that women with long-term conditions are better informed about their infant feeding options, have a more accurate expectation of barriers and facilitators to breastfeeding, and are clearer about what matters to them (Stacey, 2017).

## Conclusion

Women with autoimmune rheumatic diseases find it difficult to make well informed decisions about infant feeding and the management of their long-term condition due to a lack of information and support. Some women report feeling pressured into breastfeeding and express feelings of guilt when they are unable or choose not to breastfeed. Tailored interventions, involving healthcare professional training are required that adopt a non-judgmental and person-centred approach to supporting women with ARDs with infant feeding.

## Funding

This study was funded by a Wellcome Trust Institutional Strategic Support Fund grant. Neither the Wellcome Trust nor Health and Care Research Wales had any role in the design of the study, collection, analysis, and interpretation of data, or in preparation of this manuscript.

## Ethical approval

Ethical approval for the study, including the consent process, was granted by the Cardiff University School of Medicine Research Ethics Committee on 20/10/16.

## Declaration of Competing Interest

No conflicts of interest have been declared. Authors have not received money from infant formula or dairy companies.

## Appendix Supplementary materials

Image, application 1
