# Supplementary material for: “Nobody knows, or seems to know how rheumatology and breastfeeding works”: Women's experiences of breastfeeding whilst managing a long-term limiting condition – A qualitative visual methods study
Source: Midwifery. 2019 Nov;78:91–6. doi: 10.1016/j.midw.2019.08.002 (PMC6750183; doi:10.1016/j.midw.2019.08.002)

**Supplementary File 1: Participant sheet for women outlining what to expect during the interview**


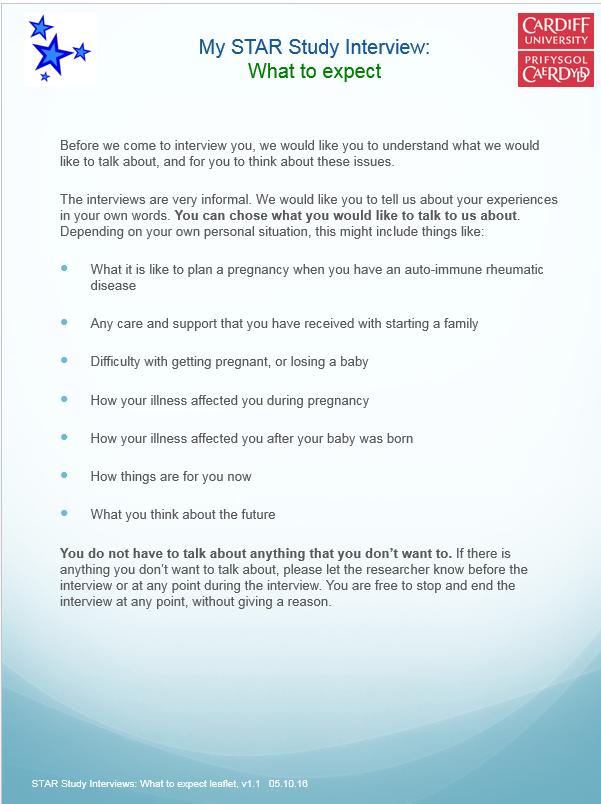


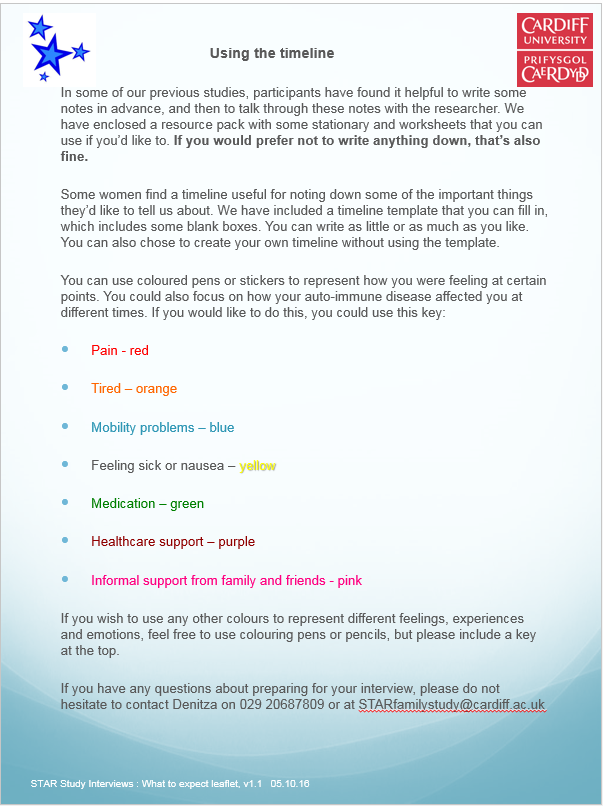

Supplement: Supplementary file 1 [file mmc1.docx]
